# Be Agreeable

**Published:** 1863-11

**Authors:** 


**Be Agreeable.**—In journeying along the Road of Life, it is a wise thing to make our fellow-travelers our friends. The way, rough as it may seem, may be pleasantly beguiled with an interchange of kindly offices and pleasant words. Suavity and forbearance are essential elements of good companionship, and no one need expect to pass pleasantly through life who does not *habitually* exercise them in his intercourse with his fellows. The Ishmaelite, whose hand is against every man, may die in a ditch without a finger being outstretched to save him. And why should we rudely jostle and shoulder our neighbors? Why tread upon each other's toes? The Christian gentleman is always careful to avoid such collisions, for courtesy and loyalty to his race are a portion of his moral and religious creed; to be loved and honored of all, his highest earthly ambition. He seeks to turn away wrath with a soft answer, and if a brawler obstinately beset his path, he steps aside to avoid him, saying, as "My Uncle Toby" said to the pertinacious fly: "Go thy ways; the world is wide enough for thee and me!"

There is another and meaner view of the subject, which we commend to the consideration of the worldly-wise and selfish. It always *pays* to be courteous, conciliating, and mild of tongue.—*Ledger.*

